# Identification of necroptosis-related subtypes and prognosis model in triple negative breast cancer

**DOI:** 10.3389/fimmu.2022.964118

**Published:** 2022-08-19

**Authors:** Shengyu Pu, Yudong Zhou, Peiling Xie, Xiaoqian Gao, Yang Liu, Yu Ren, Jianjun He, Na Hao

**Affiliations:** Department of Breast Surgery, The First Affiliated Hospital of Xi’an Jiaotong University, Xi’an, China

**Keywords:** triple negative breast cancer, necroptosis, prognosis, immune, TCGA

## Abstract

**Background:**

Necroptosis is considered to be a new form of programmed necrotic cell death, which is associated with metastasis, progression and prognosis of various types of tumors. However, the potential role of necroptosis-related genes (NRGs) in the triple negative breast cancer (TNBC) is unclear.

**Methods:**

We extracted the gene expression and relevant clinicopathological data of TNBC from The Cancer Genome Atlas (TCGA) databases and the Gene Expression Omnibus (GEO) databases. We analyzed the expression, somatic mutation, and copy number variation (CNV) of 67 NRGs in TNBC, and then observed their interaction, biological functions, and prognosis value. By performing Lasso and COX regression analysis, a NRGs-related risk model for predicting overall survival (OS) was constructed and its predictive capabilities were verified. Finally, the relationship between risk_score and immune cell infiltration, tumor microenvironment (TME), immune checkpoint, and tumor mutation burden (TMB), cancer stem cell (CSC) index, and drug sensitivity were analyzed.

**Results:**

A total 67 NRGs were identified in our analysis. A small number of genes (23.81%) detected somatic mutation, most genes appeared to have a high frequency of CNV, and there was a close interaction between them. These genes were remarkably enriched in immune-related process. A seven-gene risk_score was generated, containing *TPSG1, KRT6A, GPR19, EIF4EBP1, TLE1, SLC4A7, ESPN*. The low-risk group has a better OS, higher immune score, TMB and CSC index, and lower IC50 value of common therapeutic agents in TNBC. To improve clinical practicability, we added age, stage_T and stage_N to the risk_score and construct a more comprehensive nomogram for predicting OS. It was verified that nomogram had good predictive capability, the AUC values for 1-, 3-, and 5-year OS were 0.847, 0.908, and 0.942.

**Conclusion:**

Our research identified the significant impact of NRGs on immunity and prognosis in TNBC. These findings were expected to provide a new strategy for personalize the treatment of TNBC and improve its clinical benefit.

## Introduction

Currently, female breast cancer has become the most common cancer, and its mortality rate ranks fifth among all cancer deaths (1). About 15-20% of all newly diagnosed breast cancers are triple-negative breast cancer (TNBC), which has a worse prognosis and accounts for 5% of all cancer-related deaths (2). Due to lack ER, PR, and HER2 expression, TNBC is insensitive to endocrine therapy or anti-HER2 therapy and has limited benefit from chemotherapy and characterized by early recurrence and poor outcomes (2). There is a lack of effective treatment strategies for TNBC. Therefore, it is of great significance to find new biomarkers for optimal treatment and prediction prognosis of TNBC, which has become a continuous hot spot in breast cancer research for many years.

Necroptosis was considered a new programmed form of necrotizing cell death (3), which mainly mediated by *RIPK1, RIPK3*, and *MLKL*, and inhibited by *NEC1* (4). According to previous literature, necroptosis was implicated in the pathogenesis of neuroinflammatory diseases such as Alzheimer’s disease, Parkinson’s disease, and traumatic brain injury (5–7). In addition, it also plays an important role in carcinogenesis and has been proved strongly associated with tumor progression (8). Necroptosis can prevent tumor development, but can also promote cancer metastasis and immunosuppression by triggering an inflammatory response. This dual effect on tumors has been found in multiple cancer types (8–10). Therefore, regulating necroptosis in tumors may be an innovative and potential therapeutic strategy. There are several studies analyzed the relationship between necroptosis and breast cancer. Zheng.L et al. screened necroptosis-associated miRNAs for predicting breast cancer metastasis (11). Chen. F et al. established a risk model based on 7 necroptosis-related lncRNAs to predict breast cancer prognosis (12). However, there have been few studies have analyzed the necroptosis-related genes (NRGs) signature in TNBC.

In this study, NRGs expression data and related clinical data of TNBC patients were downloaded from The Cancer Genome Atlas (TCGA) database. First, we analyzed the expression profiles of NRGs and divided samples into two distinct necroptosis subtypes based on NRGs expression levels. Next, we identified differentially expressed genes (DEGs) based on the two necroptosis subtypes and divided patients into three gene subtypes. Then, a necroptosis-related prognostic risk model was constructed to predict overall survival (OS) of patients. We also analyzed the genetic mutation, biological process, immune landscape, and drug susceptibility et al. of NRGs within TNBC.

## Methods

### Data collection

**Figure 1:**
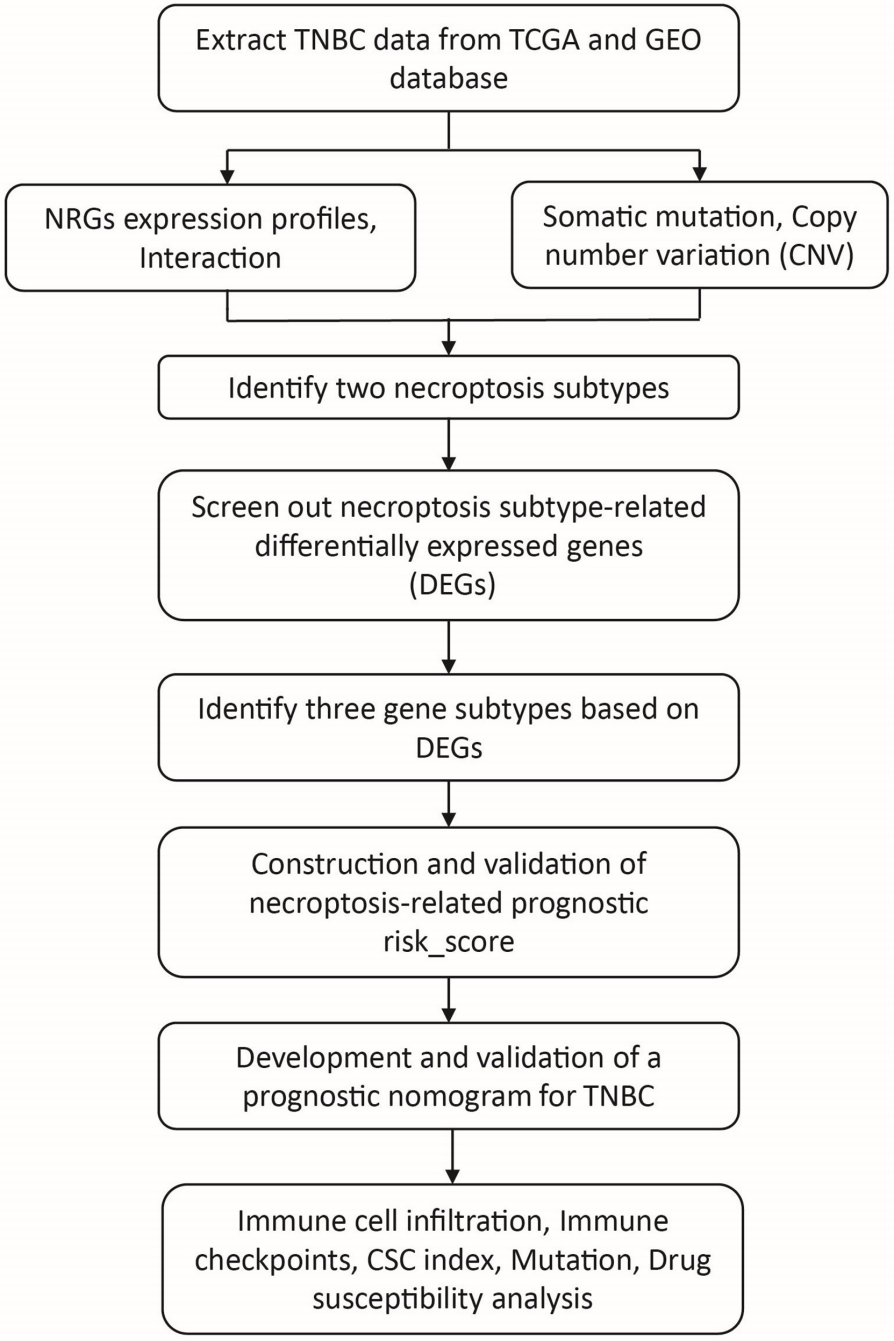
The workflow of the data analysis. TNBC, triple negative breast cancer; NRGs, necroptosis-related genes; CSC, cancer stem cell.

The analysis process of this study is shown in **Figure 1**. We search the breast cancer data by the keyword: “BRCA” in The Cancer Genome Atlas (TCGA) (https://portal.gdc.cancer.gov/) and “TNBC and survival” in the Gene Expression Omnibus (GEO) databases (https://www.ncbi.nlm.nih.gov/geo/) database and written by English. The patients meeting the following criteria were included: 1). Histologically diagnosed breast cancer; 2). Negative expression of ER, PR, and HER2; 3). Available gene expression profiling by array of homo sapiens; 4). Complete follow-up information. After excluding data with incomplete records, 275 cases with follow-up time and 155 cases with full clinicopathological information were selected (**Table S1**). We obtained the gene expression (fragments per kilobase million, FPKM), mutation, copy number variation (CNV), and relevant clinicopathological data of breast cancer from the TCGA database and the GEO database, which including four TNBC cohorts (GSE39004, GSE58812, GSE10886 and GSE97342). Moreover, we selected TNBC samples from the Metabric database (http://www.cbioportal.org/study/summary?id=brca_metabric) and GSE31519 datasets as external validation cohort. Then, we transformed the FPKM values of TCGA-BRCA into transcripts per kilobase million (TPM) for further analysis.

### Genetic mutation, CNV and consensus clustering analysis of NRGs

According to GSEA website (https://www.gsea-msigdb.org/gsea) and previous literatures (12–14), we extracted 67 necroptosis-related genes (NRGs). The summary of these 67 NRGs was listed in the **Tables S2 , S3**. The R package “maftools” and “RCircos” was used to show the genetic mutation and locations of CNV alterations of NRGs on 23 chromosomes, respectively. Based on these NRGs, we performed consensus clustering analysis to identify different necroptosis subtypes by the R package “ConsensusClusterPlus”. The overall survival (OS) of different necroptosis subtypes was assessed by the R package “survival” and “survminer”. Using the R package “Limma” to identify the DEGs between the two necroptosis subtypes. Furthermore, we observed the relationship between different necroptosis subtypes and clinicopathological characteristics, such as age and TNM stage, by the R package “pheatmap”.

### GSVA, PCA, and functional enrichment analysis

The Gene Set Variation Analysis (GSVA) was employed to investigate the difference of relevant biological process between different necroptosis subtypes. In addition, we used the single-sample gene set enrichment analysis (ssGSEA) algorithm to observe the levels of immune cell infiltration in the different necroptosis subtypes. The principal component analysis (PCA) was used to assort TNBC samples based on necroptosis subtypes. The gene ontology (GO) analysis and Kyoto Encyclopedia of Genes and Genomes (KEGG) pathways analysis was applied to identify the biological process of NRGs. The “GSEABase”, “GSVA”, “clusterProfiler”, “limma”, “org.Hs.e.g.db”, and “enrichplot” etc R packages were used in this process.

### Construction of the necroptosis-related prognostic risk model

First, the prognostic-related NRGs were identified by univariate COX regression with *P* < 0.05. Then, we divided samples into different gene clusters with the unsupervised clustering analysis according to the expression of prognostic-related NRGs. Similarly, OS and clinicopathological characteristics of different gene clusters were observed. Next, all data were randomly divided into training set and testing set in a 1:1 ratio by the “caret” R package, then established the necroptosis-related prognostic risk model in the training set. The risk model was validated in the testing set and external cohort. LASSO regression was applied to prevent over-fitting and observe the trajectory of each variable by the “glmnet” R package. Finally, the independent prognostic-related genes were screened out by multivariate COX regression analysis. We used risk-score and clinicopathological characteristics to build a nomogram by the “rms” R package. The discrimination of the model was assessed using the time-dependent area under the ROC curve (AUC) and the correction of the model was evaluated by the calibration curve. According to the median risk score in the training set, the patients were classified to low-risk group and high-risk group. Sankey diagram was made to show the cluster distribution with different risk group and survival outcomes by the “dplyr” R package.

### Tumor Immune and cancer stem cell (CSC) index Analysis

We used CIBERSORT algorithm to perform the tumor immune analysis. First, we investigated the correlation between prognostic-related genes and risk score with 22 tumor-infiltrating immune cells. Then, we calculated tumor microenvironment (TME) scores by “Estimate” R package for high- and low-risk groups, which including stromal score, immune score, and estimate score. In addition, we explored the relationship between stemness scores and risk score.

### Tumor mutation and drug susceptibility analysis

We converted the somatic mutation file extracted from the TCGA database into mutation annotation format (MAF) with the “maftools” R package and observed mutation status of samples in the high- and low-risk groups. Furthermore, we calculated the tumor mutation burden (TMB) score of two risk groups and investigated the correlation between TMB score and risk score. Finally, we used the “pRophetic” R package to calculate the semi-inhibitory concentration (IC50) values of commonly used chemotherapeutic drugs for TNBC and to compare the differences in the efficacy of chemotherapeutic drugs in high- and low-risk groups.

### qRT-PCR

Total RNA was extracted from TNBC cells (MDA-MB-231, SUM 159, and BT-549) and normal mammary epithelial cell (MCF-10A) with the RNA fast200 reagent (Fastagen Biotech; 220010). cDNA was synthesized with the StarScript II First-strand cDNA Synthesis Kit-II for qRT-PCR (Genestar). The mRNA expression levels were quantified with the SYBR-Green assays (Genestar) in the Bio-Rad CFX-96 instrument (Hercules). We processed the data through the 2-ΔΔC t strategy and selected GAPDH as an internal reference. The primer sequences used in this study were listed in **Table S4**.

### Statistical analysis

All statistical analyses were conducted with R statistical software (version 4.1.1, R Foundation for Statistical Computing, Vienna, Austria). A two-tailed *P*<0.05 was considered statistically significant.

## Results

### Genetic expression and mutation of NRGs in breast cancer

**Figure 2:**
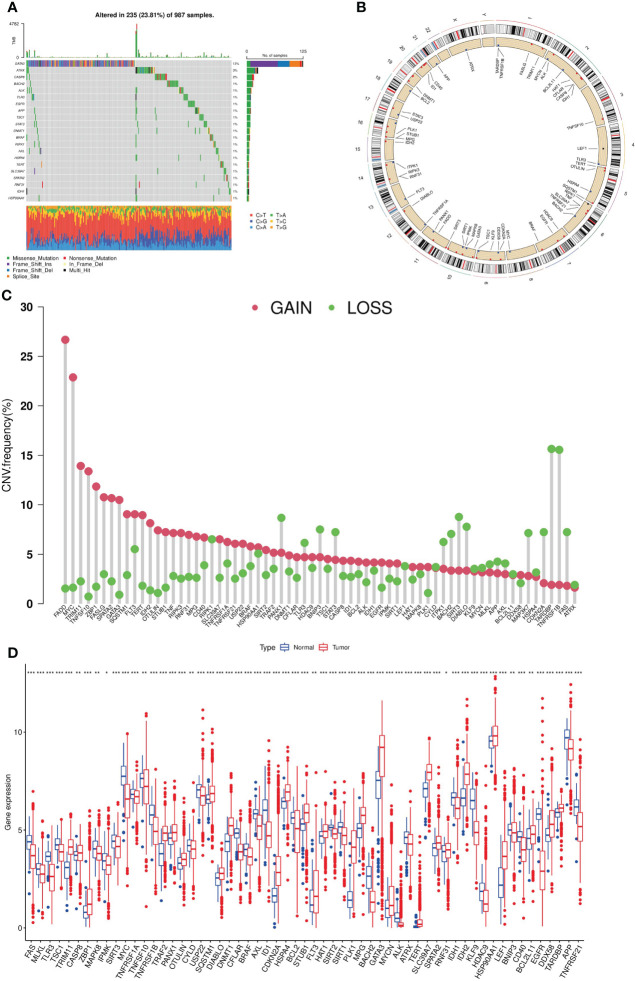
Genetic expression and mutation of NRGs in breast cancer. **(A)**. Somatic mutation frequencies of NRGs; **(B)**. The CNV locations on the 23 chromosomes of NRGs. **(C)**. Frequencies of CNV gain and loss among NRGs. **(D)**. Expression differences of NRGs in tumor tissues and normal tissues (t-test, “***”, *P* = 0.001; “**”: *P* = 0.01; “*”: *P* = 0.05). NRGs, necroptosis-related genes; CNV, copy number variation.

A total of 67 NRGs were included in this study. A pooled analysis of the incidence of somatic mutations in these 67 NRGs showed that, out of 987 breast cancer samples, 235 (23.81%) had mutations in NRGs (**Figure 2A**). Among them, *GATA3* had the highest mutation frequency (13%), and frame-shift insertion mutations accounted for the majority. Followed by *ATRX* (3%) and *CASP8* (2%), missense mutations are the most common mutations for the two genes. In addition, we investigated CNV in these NRGs and found that copy number alterations were prevalent in all 67 NRGs. Specifically, *FADD, MYC, TRIM11*, and *TNFSF10* had extensive CNV gains, while *TARDBP, TNFRSF1B, SIRT3* and *PANX1* exhibited CNV deletions (**Figure 2C**, **Table S5**). Meanwhile, we also observed gene mutation and CNV changes in the Metabric database (**Figure S1**). The CNV alterations of NRGs and their locations on the 23 chromosomes were shown in **Figure 2B** and **Table S6**. Finally, we analyzed the mRNA expression differences of NRGs in tumor tissues and normal tissues (**Figure 2D**). We found that most of the highly expressed NRGs in tumor tissues have CNV gain, such as *TRIM11, ZBP1*. However, there are also some NRGs with CNV gain, such as *FADD, FASLG*, and no significantly difference in tumor and normal tissue. This phenomenon implies that CNV can affect NRGs expression, but it is not the only influencing factor (15).

### Identification of necroptosis subtypes in TNBC

**Figure 3:**
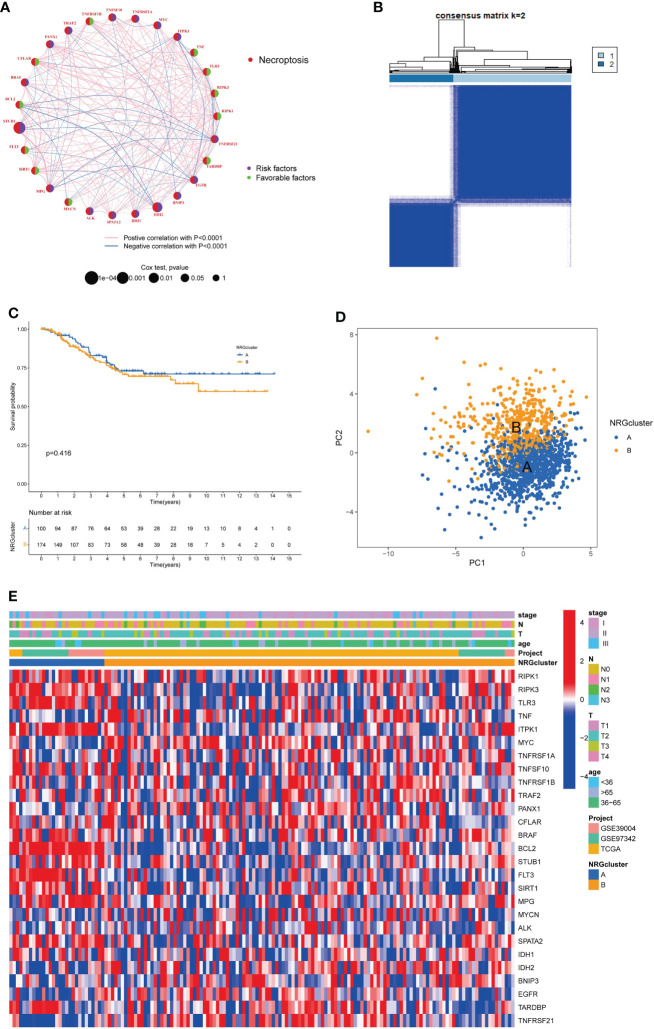
Identification of necroptosis subtypes and clinicopathological characteristics in TNBC. **(A)**. Interactions and prognostic value of NRGs; **(B)**. Consensus matrix heatmap defining two necroptosis subtypes (k = 2). **(C)**. The Kaplan-Meier analysis for OS of two necroptosis subtypes. **(D)**. The PCA analysis of two necroptosis subtypes. **(E)**. Clinicopathologic characteristics and expression levels of NRGs of two necroptosis subtypes. NRGs, necroptosis-related genes; TNBC, triple negative breast cancer; OS, overall survival; PCA, principal components analysis.

To explore the effect of NRGs on tumorigenesis, we performed COX univariate regression analysis to analyze the relationship between NRGs and breast cancer OS and plot the survival curve for the genes based on the threshold of *P*<0.05 (**Figure S2** and **Table S7**). Then, we used a necroptosis network to show these NRGs interactions, mutually regulation, and their prognostic value in breast cancer patients (**Figure 3A**). To investigate the landscape of NRGs expression in patients with TNBC, we performed the consensus clustering algorithm to categorize the samples according to the expression profiles of 67 NRGs. Our results indicated that k=2 was the best choice for dividing the entire samples into subtype A and subtype B (**Figure 3B**). The Kaplan-Meier curves showed subtype A has a better OS than type B (log-rank test, *P* = 0.416; **Figure 3C**). In addition, the PCA analysis implied a remarked difference in necroptosis transcriptional profiles between the two subtypes (**Figure 3D**). Furthermore, we compared the clinicopathological features of different subtypes of breast cancer with a heatmap (**Figure 3E**). We also add more samples from Metabric database to 369 cases for further validation (**Figure S3** and **Table S8**). The results showed that the distribution of age (*P* = 0.047) and T stage (*P* = 0.047) between two subtypes was significant.

### Characteristics of the biological behavior in necroptosis subtypes

**Figure 4:**
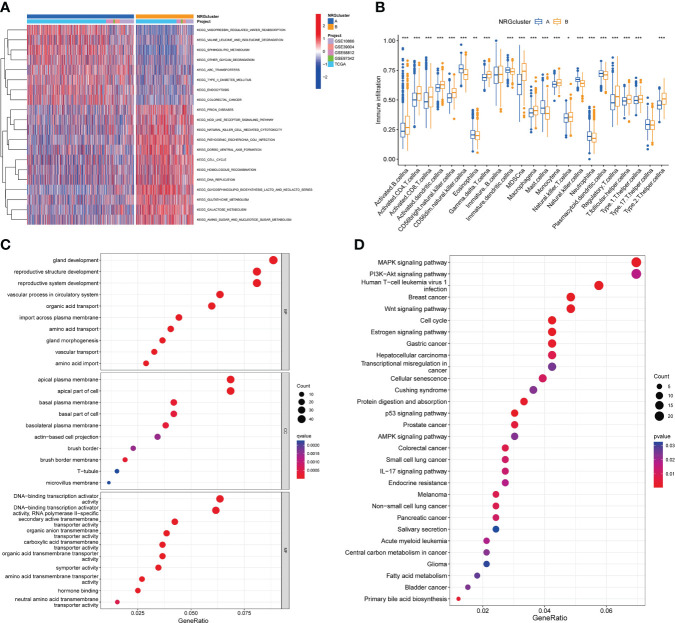
Characteristics of the biological behavior in necroptosis subtypes. **(A)**. GSVA analysis of two necroptosis subtypes. **(B)**. ssGSEA analysis of the 22 immune cell infiltration levels in two necroptosis subtypes (t-test, “***”, *P* = 0.001; “**”: *P* = 0.01; “*”: *P* = 0.05). **(C, D)**. GO **(C)** and KEGG **(D)** enrichment analyses of DEGs among two necroptosis subtypes. GSVA, Gene Set Variation Analysis; ssGSEA, single-sample gene set enrichment analysis; GO, gene ontology analysis; KEGG, Kyoto Encyclopedia of Genes and Genomes pathways analysis; DEGs, differentially expressed genes.

GSVA analysis revealed the main biological processes enriched for two subtypes and the difference were statistically significant (**Figure 4A**; **Table S9**). As shown in **Figure 4A**, subtype A was significantly enriched in vasopressin regulated water reabsorption, valine leucine and isoleucine degradation, sphingolipid metabolism, type II diabetes mellitus, endocytosis, other glycan degradation et al. biological processes. And subtype B was mainly enriched in nod like receptor signaling pathway, natural killer cell-mediated cytotoxicity, cell cycle, homologous recombination, DNA replication et al. pathways. In addition, ssGSEA algorithm showed the levels of 22 immune cell infiltration in the two subtypes (**Figure 4B**). We found that the infiltration of most immune cells was statistically different between the two subtypes. CD56dim NK cells, eosinophils, immature dendritic cells, mast cells, NK cells, neutrophils, plasmacytoid dendritic cell had significantly higher infiltration in subtype A than those in subtype B. However, subtype B had more immune cell infiltration, such as activated B cells, activated CD4 T cells, activated CD8 T cells, activated dendritic cells, CD56 bright NK cells, gamma delta T cells, MDSC cells, macrophage, Monocyte, NK T cells, regulatory T cells, T follicular helper cells et al.

Moreover, we screened out 527 necroptosis subtype-related DEGs (**Table S10**) and conducted functional enrichment analysis (**Figures 4C, D**; **Table S11**).GO analysis showed that these subtype-related DEGs were involved in biological processes such as gland development, reproductive structure and system development, apical plasma membrane, DNA-binding transcription activator activity et al. KEGG pathway analysis suggested that these NRGs were mainly related to MAPK signaling pathway, PI3K-Akt signaling pathway, human T-cell leukemia virus 1 infection, breast cancer, Wnt signaling pathway, cell cycle, transcriptional misregulation in cancer et al.

### Identification of gene subtypes in TNBC based on DEGs

**Figure 5:**
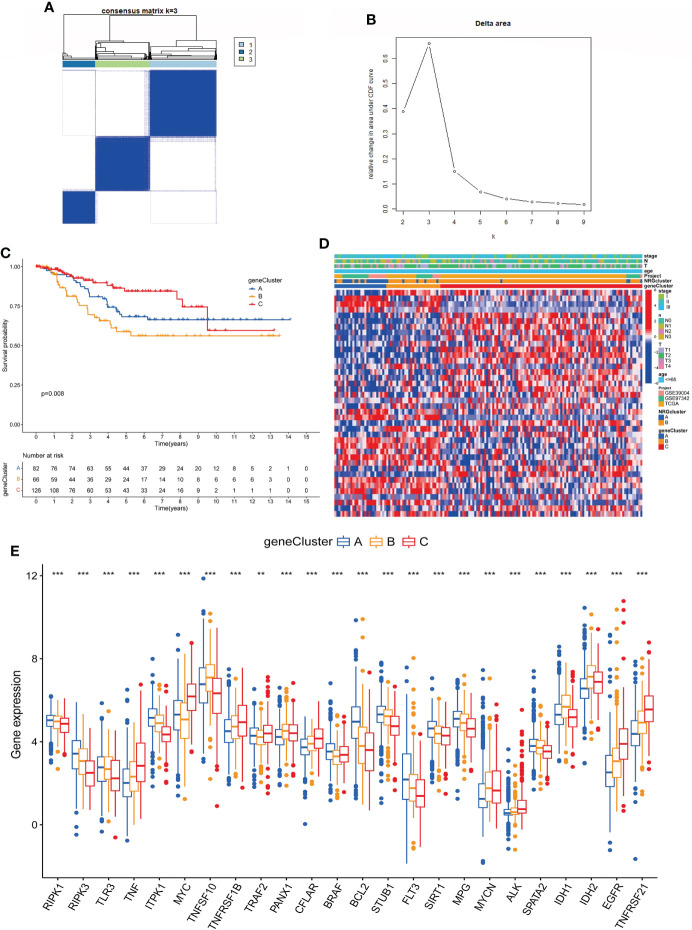
Identification of gene subtypes based on DEGs. **(A, B)**. Consensus matrix heatmap defining three gene subtypes (k = 2). **(C)**. The Kaplan-Meier analysis for OS of three gene subtypes. **(D)**. Clinicopathologic characteristics distribution of three gene subtypes and two necroptosis subtypes. **(E)**. Expression of NRGs in three gene subtypes (t-test, “***”, *P* = 0.001; “**”: *P* = 0.01; “*”: *P* = 0.05). DEGs, differentially expressed genes; OS, overall survival; NRGs, necroptosis-related genes.

We performed univariate COX regression for 527 DEGs to analyze their prognostic value in TNBC, and obtained 40 genes according to *P* < 0.05 for further analysis (**Table S12**). Then, based on these prognosis-related genes, we used a consensus clustering algorithm to divide the cohort into three gene subtypes (**Figures 5A, B**). Kaplan–Meier survival analysis suggested that the OS among the three gene subtypes were significant (*P* = 0.008), and gene cluster B having the worst prognosis (**Figure 5C**). Then, we analyzed the distribution of clinicopathological features in each gene cluster with a heatmap (**Figure 5D**). Finally, the expression of NRGs in each gene cluster was analyzed, and the results showed that the expression of NRGs was significantly different (**Figure 5E**).

### Construction of necroptosis-related prognostic risk_score

**Figure 6:**
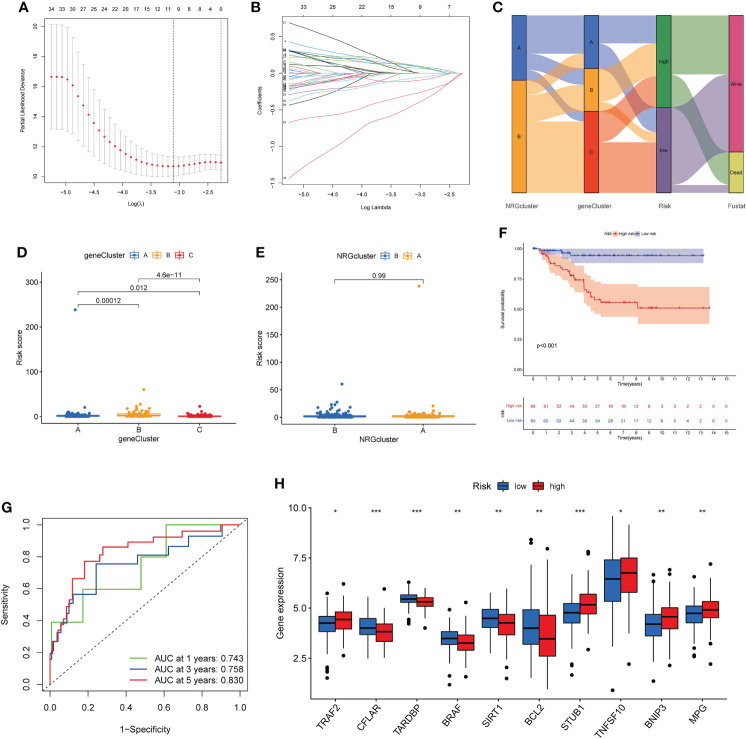
Construction of necroptosis-related prognostic risk_score. **(A, B)**. Lasso regression analysis on the prognosis-related genes. **(C)**. Sankey diagram of samples distribution in two necroptosis subtypes, three gene subtypes and two risk_score groups. **(D, E)**. Difference of risk_score among the three gene subtypes **(D)** and two necroptosis subtypes **(E)**. **(F)**. The Kaplan-Meier analysis for OS of two risk groups. **(G)**. ROC curves to predict 1-, 3-, and 5-year OS according to the risk _score in the training cohort. **(H)**. Expression of NRGs in two risk groups (t-test, “***”, *P* = 0.001; “**”: *P* = 0.01; “*”: *P* = 0.05). OS, overall survival; NRGs, necroptosis-related genes.

Based on 40 prognosis-related genes, we constructed a necroptosis-related prognostic risk model (**Figure 6**). First, we randomly divided the cohort into training and testing sets in a 1:1 ratio, and then selected the optimum prognostic factors by Lasso regression and COX multivariate regression analysis. According to Lasso regression, 11 OS-related genes were retained by minimum partial likelihood deviance (**Figures 6A, B**). COX multivariate regression analysis was performed based on these 11 OS-related genes, and finally, we screened out 7 genes (*TPSG1, KRT6A, GPR19, EIF4EBP1, TLE1, SLC4A7, ESPN*) for constructing risk model. The risk_score was constructed as follows:

Risk_score = (0.2668* expression of *TPSG1*) + (0.1289* expression of *KRT6A*) + (-1.0792* expression of *GPR19*) + (0.3827* expression of *EIF4EBP1*) + (0.4732* expression of *TLE1*) + (-0.6097* expression of *SLC4A7*) + (0.2877 * expression of *ESPN*).

According to the median risk_score, the patients were classified as low-risk groups (n=132) and high-risk groups (n=142). Sankey diagram showed the distribution of samples in two necroptosis clusters, three gene clusters and two risk_score groups (**Figure 6C**). We found that risk_score was significantly different between three gene clusters (**Figure 6D**). The cluster B had the highest score, followed by cluster A, and cluster C had the lowest score. The difference of risk_score among the two necroptosis clusters was showed in **Figure 6E**. The Kaplan–Meier survival analysis of the two risk groups declared that the patients in the low-risk group could obtain significantly better OS than those in the high-risk group (log-rank test, *P*<0.001; **Figure 6F**). Furthermore, the 1-, 3- and 5-year AUC value of risk_score model were 0.743, 0.758, 0.830 in the training cohort (**Figure 6G**). The 1-, 3- and 5-year AUC value in the entire cohort were 0.736, 0.737, 0.718 and in the testing cohort were 0.728, 0.718, and 0.613, respectively (**Figure S4**). Moreover, we also validated the risk_score model in the GSE31519 dataset (**Figure S5**). Finally, we investigated differences in the expression levels of NRGs within the two risk groups (**Figure 6H**).

### Development and validation of a prognostic nomogram for TNBC

**Figure 7:**
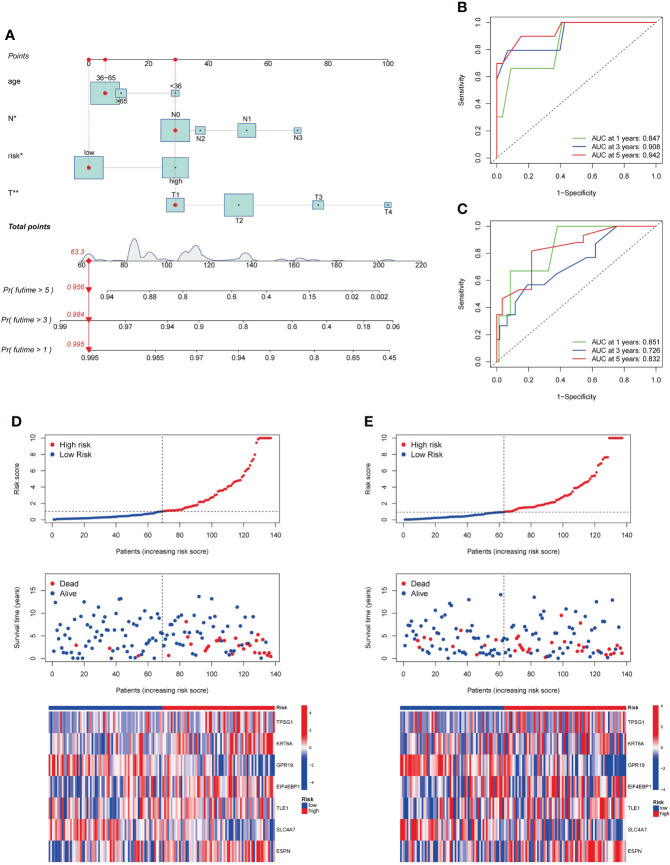
Development and validation of a prognostic nomogram. **(A)**. Nomogram for predicting OS of TNBC. **(B, C)**. ROC curves to predict 1-, 3-, and 5-year OS according to the nomogram in the training cohort **(B)** and testing cohort **(C)**. **(D, E)**. Ranked dot, scatter plots, and heatmap showing the risk_score distribution, patient survival status and gene expression in the in the training cohort and testing cohort. OS, overall survival.

To improve clinical practicability, we added clinicopathological parameters, including age, stage_T and stage_N, to the above prognostic risk model to construct a more comprehensive nomogram for predicting OS of TNBC (**Figure 7A**). It was verified that the model had good discrimination. In the training set, the AUC values for 1-, 3-, and 5-year were 0.847, 0.908, and 0.942, respectively (**Figure 7B**). In the testing set, the AUC values for 1-, 3-, and 5-year were 0.851, 0.726, and 0.832, respectively (**Figure 7C**). The calibration curve suggested that the model had good correction ability (**Figure S6**). Scatter plot of the risk distribution showed that survival time decreased and mortality increased with increasing of the risk score (**Figures 7D, E**). Finally, we validated the performance of the nomogram in the Metabric cohort (**Figure S7**).

### Characteristics of the TME in the high- and low-risk groups

**Figure 8:**
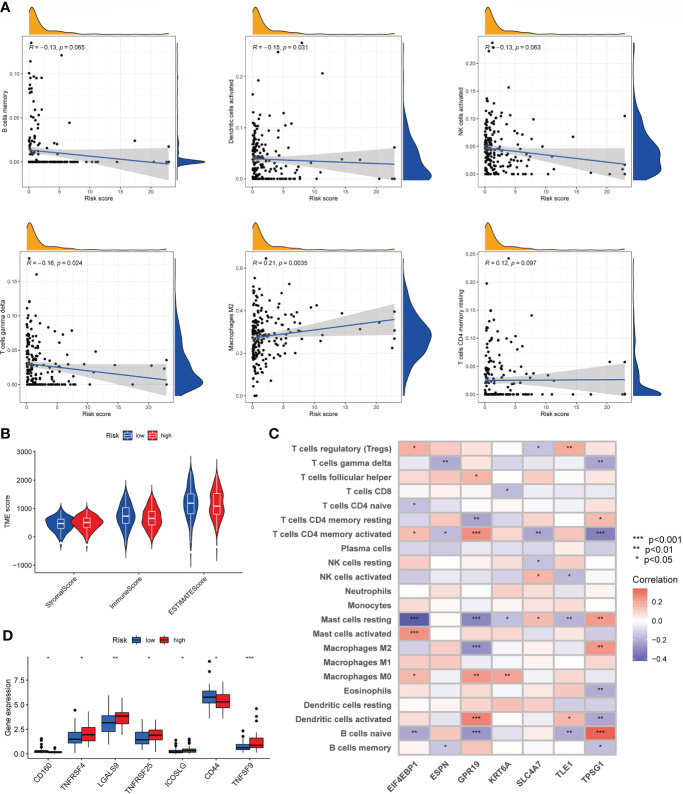
Characteristics of the TME and immune checkpoint among two risk_score groups. **(A)**. Association between risk_score and immune cell infiltration; **(B)**. Association between risk _score and both immune and stromal scores. **(C)**. Association between the abundance of immune cells and seven genes in the risk_score model. **(D)**. Expression of immune checkpoints in the high and low-risk groups. TME, tumor microenvironment.

We evaluated the association between risk_score and immune cell infiltration with the CIBERSORT algorithm. As **Figure 8A** shown, risk_score was negatively associated with memory B cells, activated dendritic cells, activated NK cells, and gamma delta T cells, and was positively correlated with M2 macrophages, resting memory CD4+ T cells. In addition, low risk_score was also associated with higher immune score compared to high risk_score (**Figure 8B**). Then, we investigated the relationship of seven genes in the model with immune cells and found that these genes were significantly associated with most immune cells (**Figure 8C**). Finally, we observed the expression of immune checkpoints among two risk group. As **Figure 8D** shown, some immune checkpoints were differentially expressed in the two groups.

### CSC index, mutation and drug susceptibility analysis

**Figure 9:**
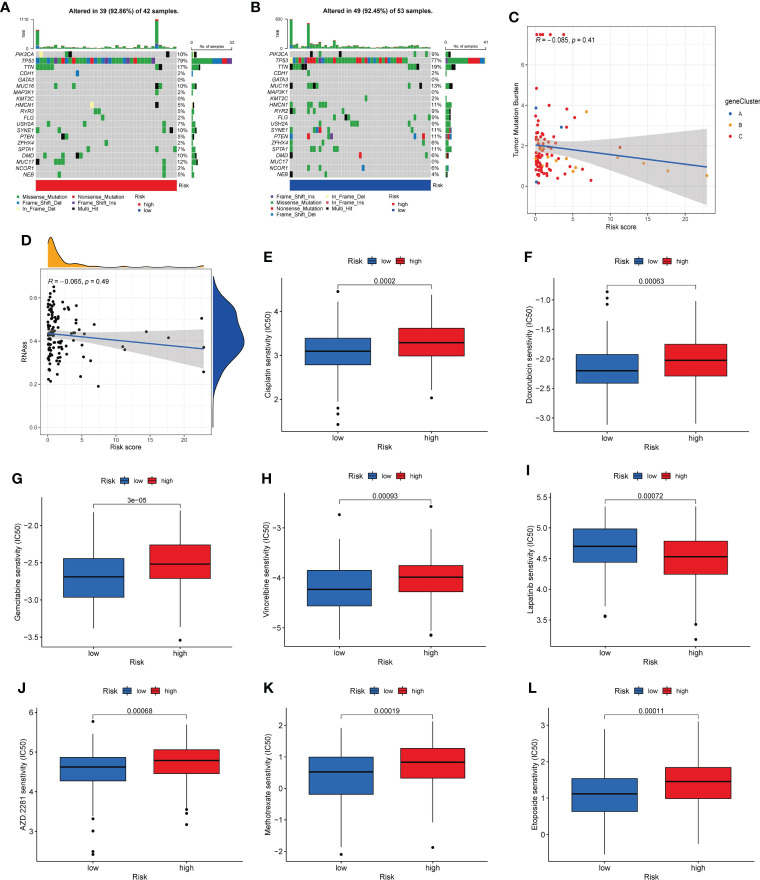
Mutation, CSC index, and drug susceptibility analysis among two risk_score groups. **(A, B)**. The waterfall plot of somatic mutation features in the high-risk group **(A)** and low-risk group **(B)**. **(C)**. Correlation between risk_score and TMB. **(D)**. Correlation between risk_score and CSC index. **(E-L)**. Correlation between risk_score and drug sensitivity. CSC, cancer stem cell; TMB, tumor mutation burden.

We analyzed the somatic mutations in the high- and low-risk groups. The gene with the highest mutation frequency in the two risk groups was *TP53*. We found that some genes were mutated more frequently in the low-risk group than in the high-risk group, such as *PTEN* and *HMCN1*. Conversely, *MUC17* was mutated more frequently in the high-risk group than in the low-risk group (**Figures 9A, B**). Then, the TMB was negatively corrected with risk_score (**Figure 9C**). Furthermore, the potential correlations between CSC index values and risk_score was also assessed. According to **Figure 9D**, risk_score was negatively correlated with CSC index, meaning that TNBC cells with lower risk_score had more obvious stem cell properties. Finally, we assessed the susceptibility of TNBC patients in the high- and low-risk groups to some common therapeutic agents. As **Figures 9E–L** shown, most therapeutic drugs had lower IC50 value in the low-risk group, such as cisplatin, doxorubicin, gemcitabine and vinorelbine et al, whereas lapatinib had a lower IC50 value in the high-risk group.

### Validation of the expression levels of the seven NRGs in the risk model

**Figure 10:**
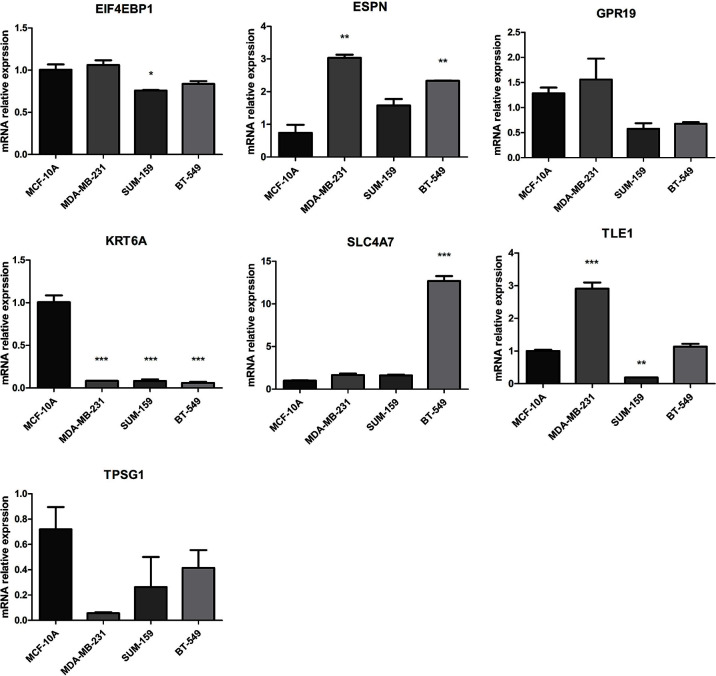
The mRNA expression of 7 prognosis related necroptosis-genes in TNBC cells and normal mammary epithelial cell by RT-PCR (t-test, “***”, *P* = 0.001; “**”: *P* = 0.01; “*”: *P* = 0.05).

**Figure 11:**
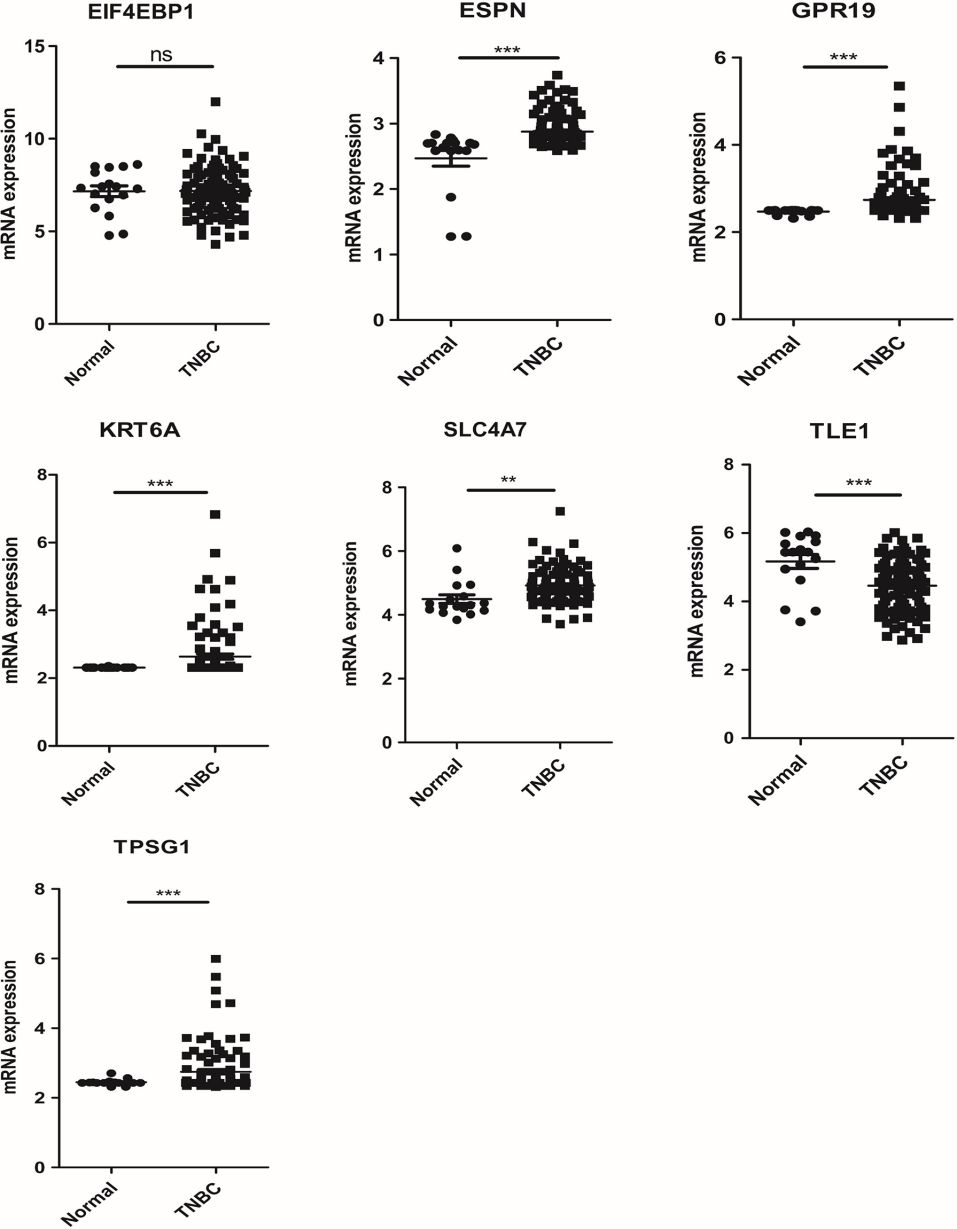
The mRNA expression of 7 prognosis related necroptosis-genes in GEO database from TNBC patients and corresponding normal tissues (t-test, “***”, *P* = 0.001; “**”: *P* = 0.01; “*”: *P* = 0.05).

The mRNA expression of seven prognosis related necroptosis-genes in TCGA database was showed in **Table S13**. In addition, we measured the expression levels of seven prognostic genes in four TNBC cells and one normal mammary epithelial cell by RT-qPCR. As shown in **Figure 10**, the expression levels of ESPN, SLC4A7 et al. were upregulated while those of TPSG1 and KRT6A were downregulated obviously in TNBC cells compared with MCF-10A. Moreover, we contrasted the expression level of these genes in TNBC patient’s tissues and corresponding normal tissues in the GSE42568 dataset (**Figure 11**, **Table S14**), the results showed that ESPN, GPR19, KRT6A, SLC4A7, TPSG1 were significantly upregulated in TNBC patients, while TLE1 was dramatically downregulated in TNBC patients. The prognostic value of seven necroptosis-related genes was summarized in the **Table S15**.

## Discussion

This is the first study, to our knowledge, to identify and comprehensively summarize the NRGs somatic mutation, expression level, CNV, immune infiltration, TME, CSC, drug sensitivity et al. in TNBC. Based on the 67 NRGs, we divided the samples into two necroptosis subtypes, and subtype A with better OS. The biological process and immune cell infiltration of the two subtypes were analyzed by GSVA and GSEA, and the results suggested that subtype B was mainly enriched in nod like receptor signaling pathway, natural killer cell-mediated cytotoxicity, cell cycle, homologous recombination, DNA replication et al. In addition, subtype B has more immune cell infiltration than subtype A. Based on the DEGs between the two necroptosis subtypes, we identified three gene subtypes and constructed a risk_score model for predicting OS in TNBC. In order to improve the clinical utility of the model, we added clinicopathological features to the model to construct a nomogram and verified its predictive performance. The prognosis, mutation, TME, CSC index and drug sensitivity of patients with low-risk and high-risk NRG scores were significantly different. Our findings revealed that NRGs could be used to assess prognosis significance and immunotherapy response in TNBC.

Breast cancer was thought to be immune cold, however, TNBC has been shown to have an immunocompetent subtype that could benefit from immunotherapy (16). The specific role of necroptosis on tumor growth by affecting tumor immune environment is still unclear. On the one hand, necroptosis can activate dendritic cells and CD8+ T lymphocytes by releasing various inflammatory cytokines, thereby inducing a strong immune response and enhancing anti-tumor immunity (10); on the other hand, immune inflammatory cells recruited by necroptosis can promote angiogenesis, tumor cell proliferation, and accelerate cancer metastasis (17, 18). In our immune analysis, the low-risk group had higher immune score compared to the high-risk group. In addition, risk_score was negatively correlated with tumor-killing immune cells such as NK cells, and positively correlated with immunosuppressive cells such as M2 macrophages. The M2 macrophages (M2-type tumor associated macrophages, TAMs) was the most common type of tumor microenvironment (TME), which played a role in inhibiting immune response in TME. Currently, inducing macrophages’ polarization to the M2 phenotype as therapeutic targets and screening the key molecular(s) modulators targeting macrophages’ polarization to the M2 phenotype could be another promising treatment strategy for cancers. For instance, RIPK1 (one of our 67 NRGs, also a common NRGs) is heavily expressed by TAMs in pancreatic cancer wherein RIPK1 facilitates TAMs-driven immunosuppression (19), and the pharmacological inhibitors of RIPK1 in pancreatic cancer patients (with or without combination with anti-PD1 immunotherapy) with the aim to ameliorate the immunosuppressive tumor milieu (20). Thus, we believe a detailed tumor tissue analyses *via* exploring key NRGs that can simultaneously capture the cells-of-origin for pro-necroptotic genes will be instrumental in solving some of treatment strategy for cancers, which also provide new clues and ideas for further research on its role in TNBC.

In decades, immune checkpoint blockers (ICBs)-based immunotherapies have allowed oncologists to anticipate tumor curative strategies, however, not all cancer patients durably respond to ICBs duo to the resistance to ICBs. To overcome these problems, prediction model or combination of ICBs with key NRGs are being urgently need to study. Snyder et al. endeavors aim to decipher whether necroptosis can successfully synergize with ICBs to create new immunotherapy (21). In mice, inducing spontaneous necroptosis in subcutaneous murine tumors by overexpressing MLKL mRNA synergized efficiently with anti-PD1 immunotherapy to elicit potent anti-tumor immunity (22). In our study, the expression level analysis of immune checkpoints showed that some immune checkpoints, such as *TNFSF9, TNFRSF4* (*OX40*)*, TNFRSF25*, and *LGALS9* were highly expressed in the high-risk group. *TNFRSF4* (*OX40*) (also a classical necroptosis related gene) was members of the TNF receptor superfamily (*TNFRSF*), proved to have an anti-tumor and regulate the function of immune cells function (23, 24). Moreover, we found that *CD44* was highly expressed in the low-risk group. *CD44* is a key regulator of *PD-L1* expression in TNBC, and it could indirectly promote cancer cell proliferation and immune evasion through mediated *PD-L1* expression (25). These diverse mechanistic studies highlight the putative benefits of combining ICBs with key necroptosis gene/molecular in cancer immunotherapy context and our necroptosis-related prognostic risk model targeting ICBs provide new clues and ideas for further research on more tumor samples.

In addition to immune cell infiltration, tumor mutation burden is also a potential biomarker for predicting treatment and prognosis in multiple tumors (26–28). According to previous reports, *TP53* is the most commonly mutated gene in TNBC (29, 30), and patients with *TP53* mutation exhibit favorable immunotherapy response profile (31). In our analysis, *TP53* had the highest mutation frequency in the two risk groups, which is consistent with the results in the above literatures. *TTN* mutations are also frequently detected in TNBC, and studies have suggested that *TTN* mutations could increase TMB and improve the objective response to immune checkpoint blockade therapy (32). Moreover, patients with *TTN* mutations have higher progression-free survival (PFS) or OS than wild-type patients (33). TMB is strongly corrected to tumor treatment efficacy and prognosis, specifically high TMB produces better survival (34–36). In our analysis, the low-risk group had higher TMB and better survival, the *P*-value is not statistically significant may be due to our small sample size.

It has been shown that TNBC exhibits more CSC features than other breast cancer subtypes, which may contribute to its high invasiveness and susceptibility to metastases (37, 38). In addition, previous studies have shown that CSCs were the source of chemotherapy resistance in TNBC (39, 40). In our study, the necroptosis risk_score was negatively associated with the CSC index. Moreover, drug sensitivity analysis revealed the IC50 values of common chemotherapy drugs such as cisplatin, doxorubicin, gemcitabine, etc. in the low-risk group were lower than those in the high-risk group. Targeted CSC therapy could potentially prevent metastasis and thus TNBC survival (41). We speculated that the effect of CSC on tumor growth and metastasis might be related to necroptosis.

This study has several limitations. First, our study is a retrospective study, which inevitably has selection bias. Second, the number of TNBC cases is limited and some important clinical information are lacking, such as surgery and neoadjuvant therapy, which are also important factors affecting the prognosis of TNBC. Finally, our findings require further validation of external clinical data, as well as *in vivo* and *in vitro* experiments.

## Conclusion

This study comprehensively analyzed the NRGs mutation, CNV, expression profile, and their impact on tumor immune infiltration, CSC index, drug sensitivity, and prognosis values. We constructed a NRGs-related prognostic model, indicating the potential influence of NRGs in immunotherapy and targeted therapy. These finding are expected to provide a new strategy for personalize the treatment of TNBC and improve its clinical benefit.

## Data availability statement

Publicly available datasets were analyzed in this study. This data can be found here: https://portal.gdc.cancer.gov/, https://www.ncbi.nlm.nih.gov/geo/, http://www.cbioportal.org/study/s%20ummary?id=brca_metabric. The accession number(s) can be found in the article/**Supplementary Material**.

## Author contributions

SP, conceptualization, methodology, and writing original draft. YZ, formal analysis, investigation, and validation. PX and XG, software and visualization. YL and YR, data curation and resources. JH, writing- reviewing and editing, supervision. NH, conceptualization, writing - review and editing. All authors contributed to the article and approved the submitted version.

## Funding

This review was funded by The National Natural Science Foundation of China (NSFC; no. 26 82003183, to NH).

## Conflict of interest

The authors declare that the research was conducted in the absence of any commercial or financial relationships that could be construed as a potential conflict of interest.

## Publisher’s note

All claims expressed in this article are solely those of the authors and do not necessarily represent those of their affiliated organizations, or those of the publisher, the editors and the reviewers. Any product that may be evaluated in this article, or claim that may be made by its manufacturer, is not guaranteed or endorsed by the publisher.

## Supplementary material

Supplementary Figure 1 Somatic mutation **(A)** and frequencies of CNV **(B)** of NRGs in breast cancer from the Metabric database. CNV, copy number variation; NRGs, necroptosis-related genes.

Supplementary Figure 2 The Kaplan-Meier analysis for OS of NRGs in TNBC. NRGs, necroptosis-related genes.

Supplementary Figure 3 Clinicopathologic characteristics and expression levels of NRGs of two necroptosis subtypes after add the data from the Metabric database. NRGs, necroptosis-related genes.

Supplementary Figure 4 The Kaplan-Meier curves and ROC curves to predict 1-, 3-, and 5-year OS according to the risk _score in the entire cohort **(A, C)** and testing cohort **(B, D)**.

Supplementary Figure 5 The validation of risk _score in the GSE31519 dataset. **(A)**. ROC curves to predict 1-, 3-, and 5-year EFS according to the risk _score; **(B)**. The Kaplan-Meier analysis for EFS of two risk groups. **(C)**. Ranked dot, scatter plots, and heatmap showing the risk_score distribution, patient survival status and gene expression. EFS, event-free survival.

Supplementary Figure 6 The calibration curves of nomogram in the training cohort **(A)** and testing cohort **(B)**.

Supplementary Figure 7 The validation of nomogram in the Metabric database. **(A)**. ROC curves to predict 1-, 3-, and 5-year OS according to the nomogram; **(B)**. The calibration curves of nomogram. **(C)**. Ranked dot, scatter plots, and heatmap showing the risk distribution, patient survival status and gene expression. OS, overall survival.
