# Supplementary figures and images for: Identification of necroptosis-related subtypes and prognosis model in triple negative breast cancer
Source: Front Immunol. 2022 Aug 19;13:964118. doi: 10.3389/fimmu.2022.964118 (PMC9437322; doi:10.3389/fimmu.2022.964118)

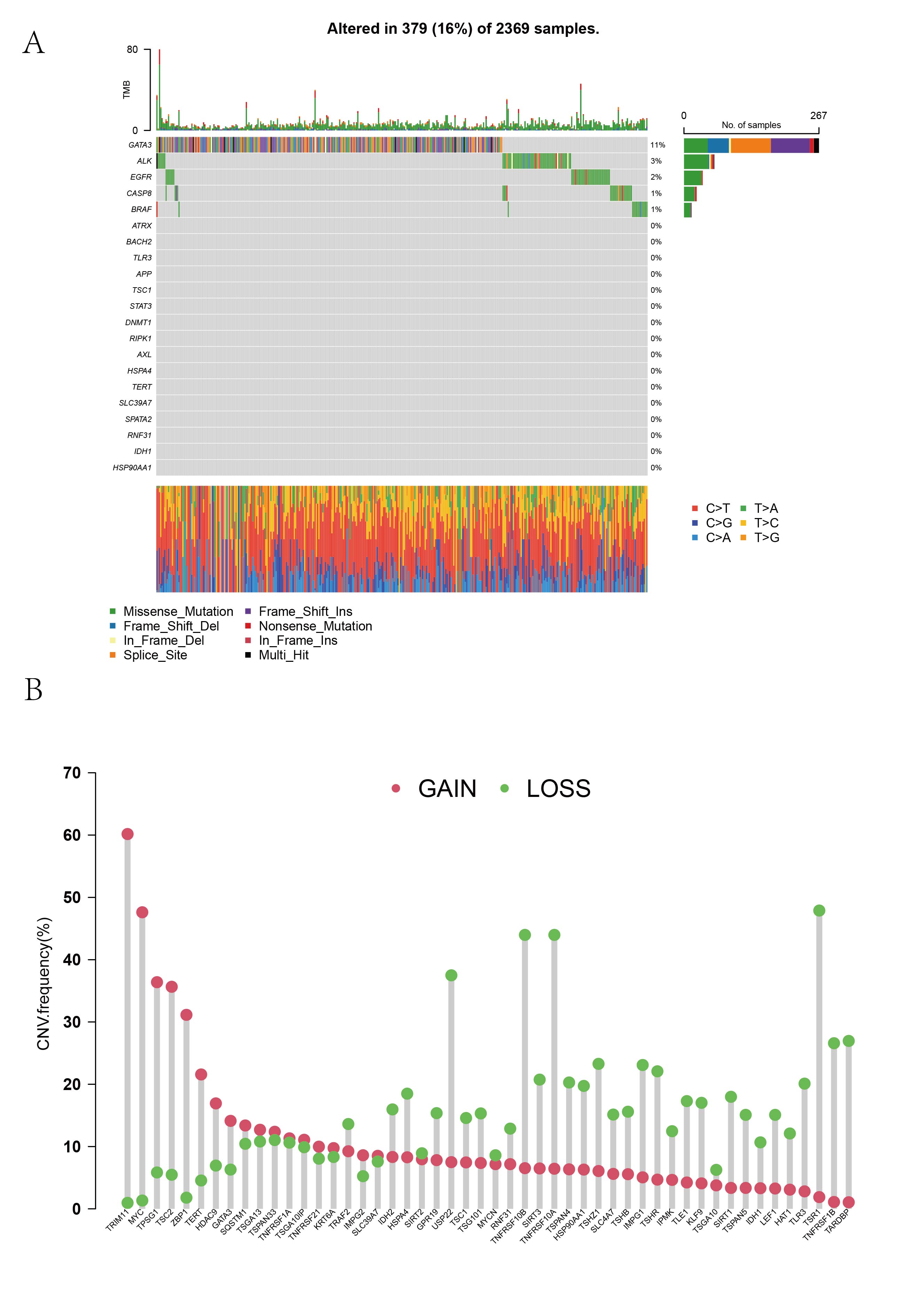

Supplement: Supplementary Figure 1 — Somatic mutation (A) and frequencies of CNV (B) of NRGs in breast cancer from the Metabric database. CNV, copy number variation; NRGs, necroptosis-related genes. [file Image_1.jpeg]

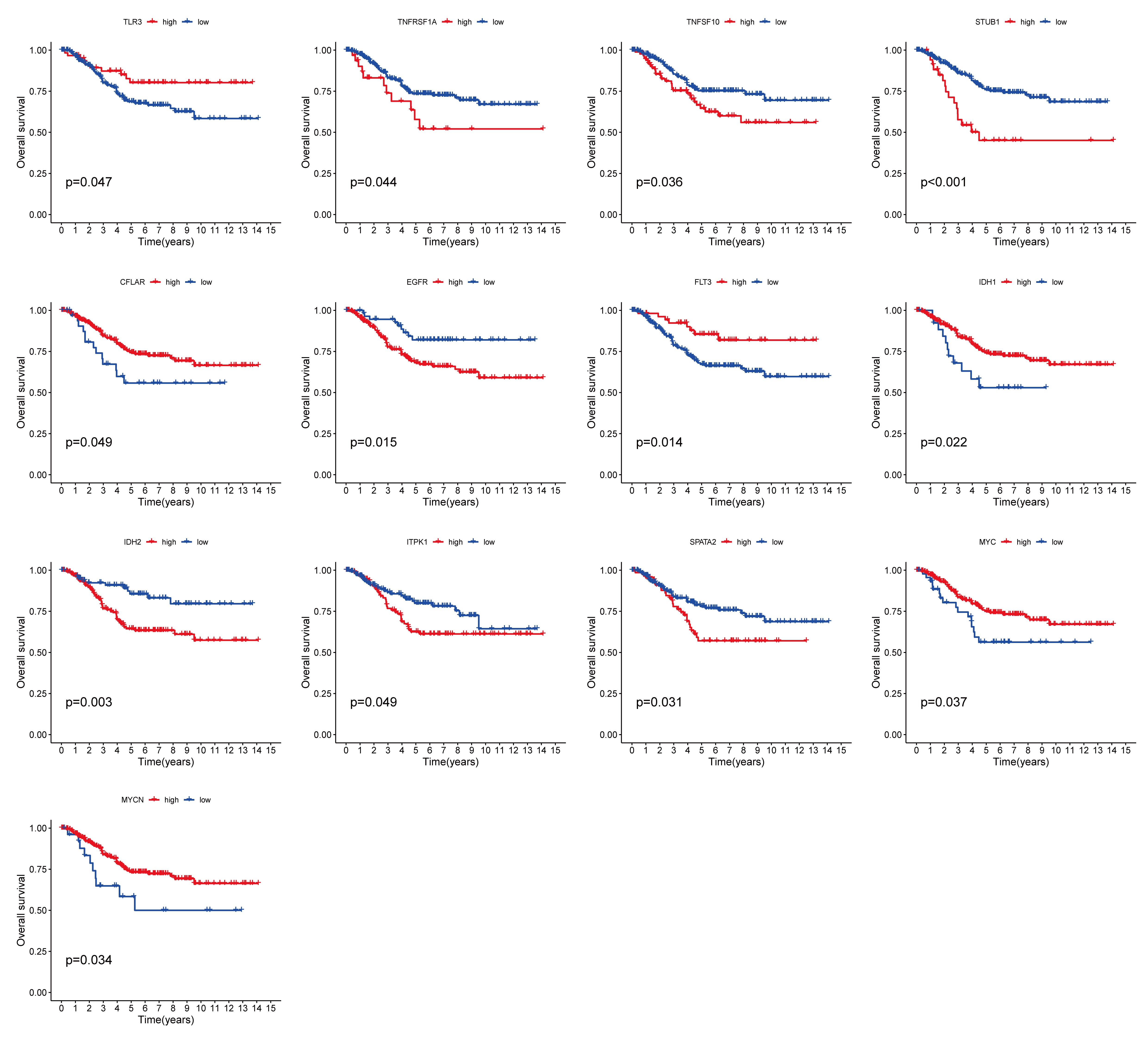

Supplement: Supplementary Figure 2 — The Kaplan-Meier analysis for OS of NRGs in TNBC. NRGs, necroptosis-related genes. [file Image_2.jpeg]

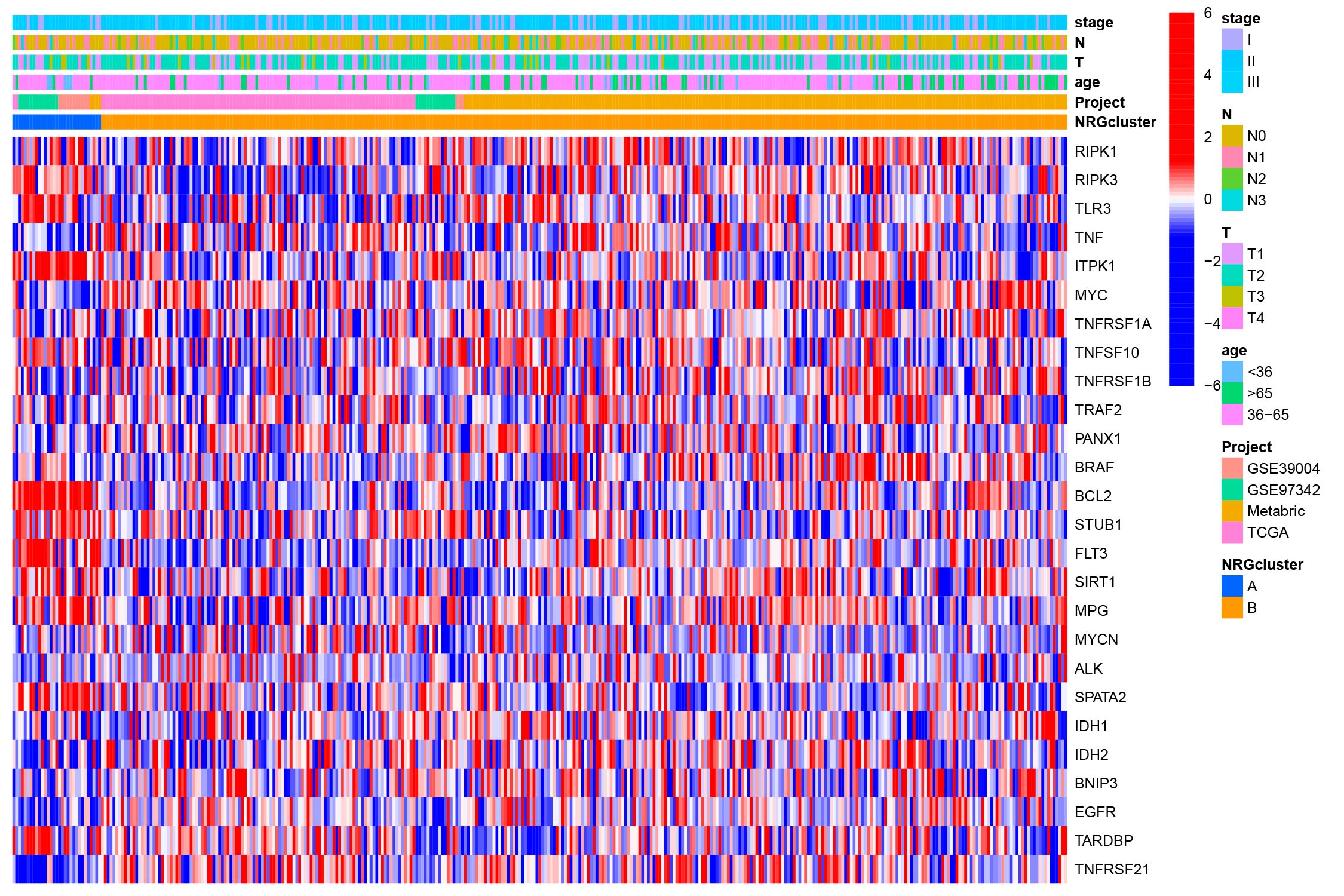

Supplement: Supplementary Figure 3 — Clinicopathologic characteristics and expression levels of NRGs of two necroptosis subtypes after add the data from the Metabric database. NRGs, necroptosis-related genes. [file Image_3.jpeg]

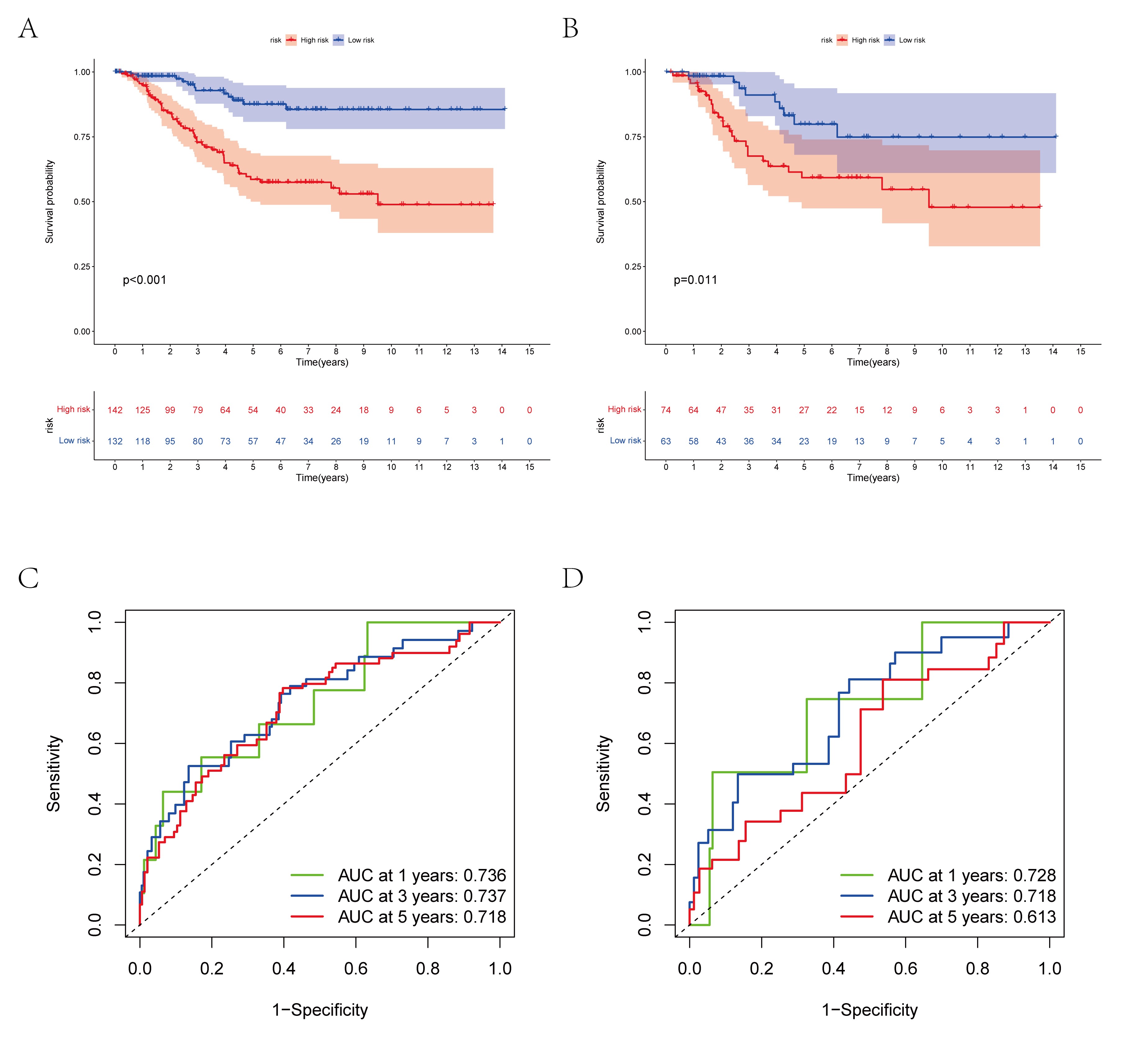

Supplement: Supplementary Figure 4 — The Kaplan-Meier curves and ROC curves to predict 1-, 3-, and 5-year OS according to the risk _score in the entire cohort (A, C) and testing cohort (B, D). [file Image_4.jpeg]

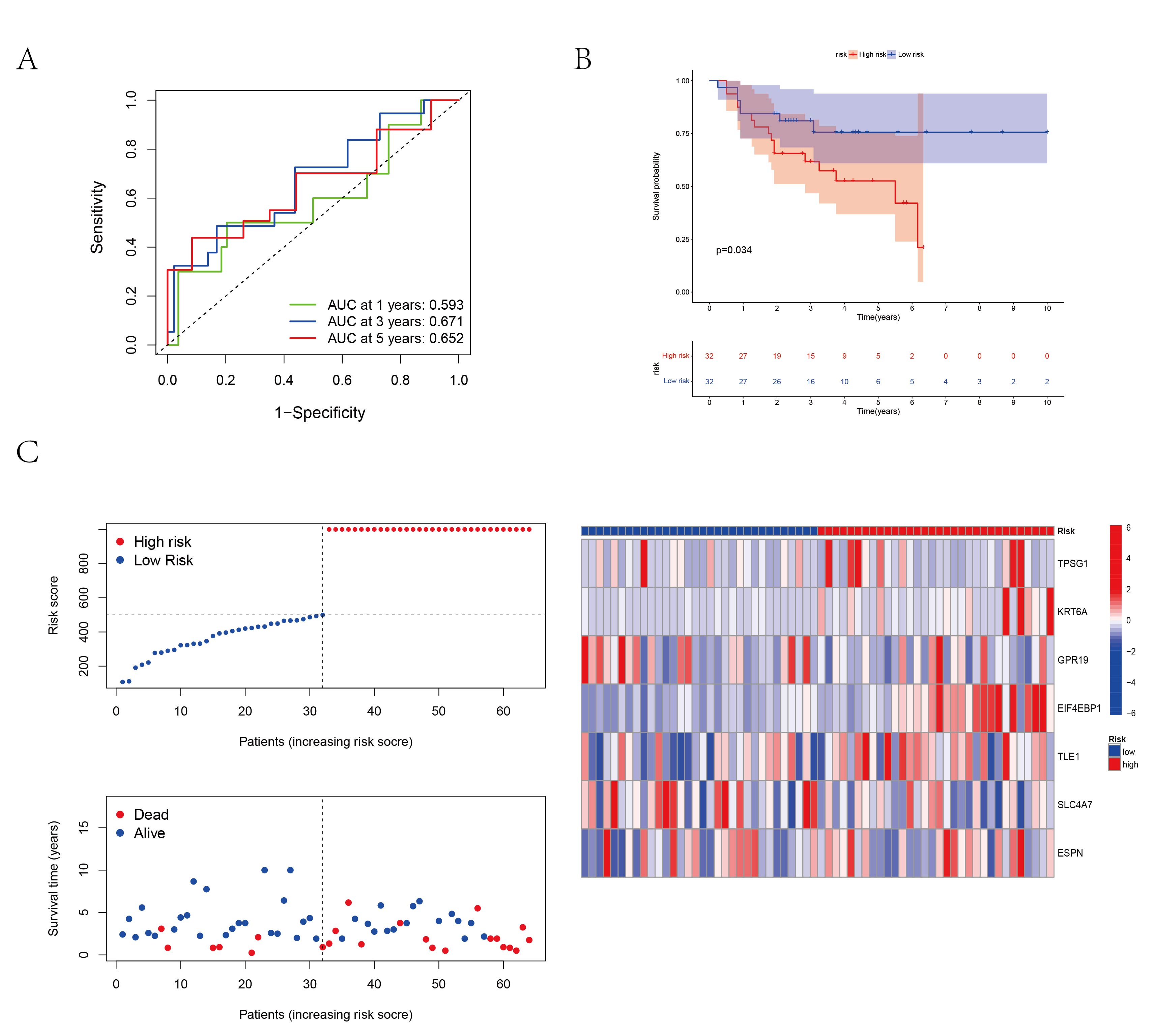

Supplement: Supplementary Figure 5 — The validation of risk _score in the GSE31519 dataset. (A). ROC curves to predict 1-, 3-, and 5-year EFS according to the risk _score; (B). The Kaplan-Meier analysis for EFS of two risk groups. (C). Ranked dot, scatter plots, and heatmap showing the risk_score distribution, patient survival status and gene expression. EFS, event-free survival. [file Image_5.jpeg]

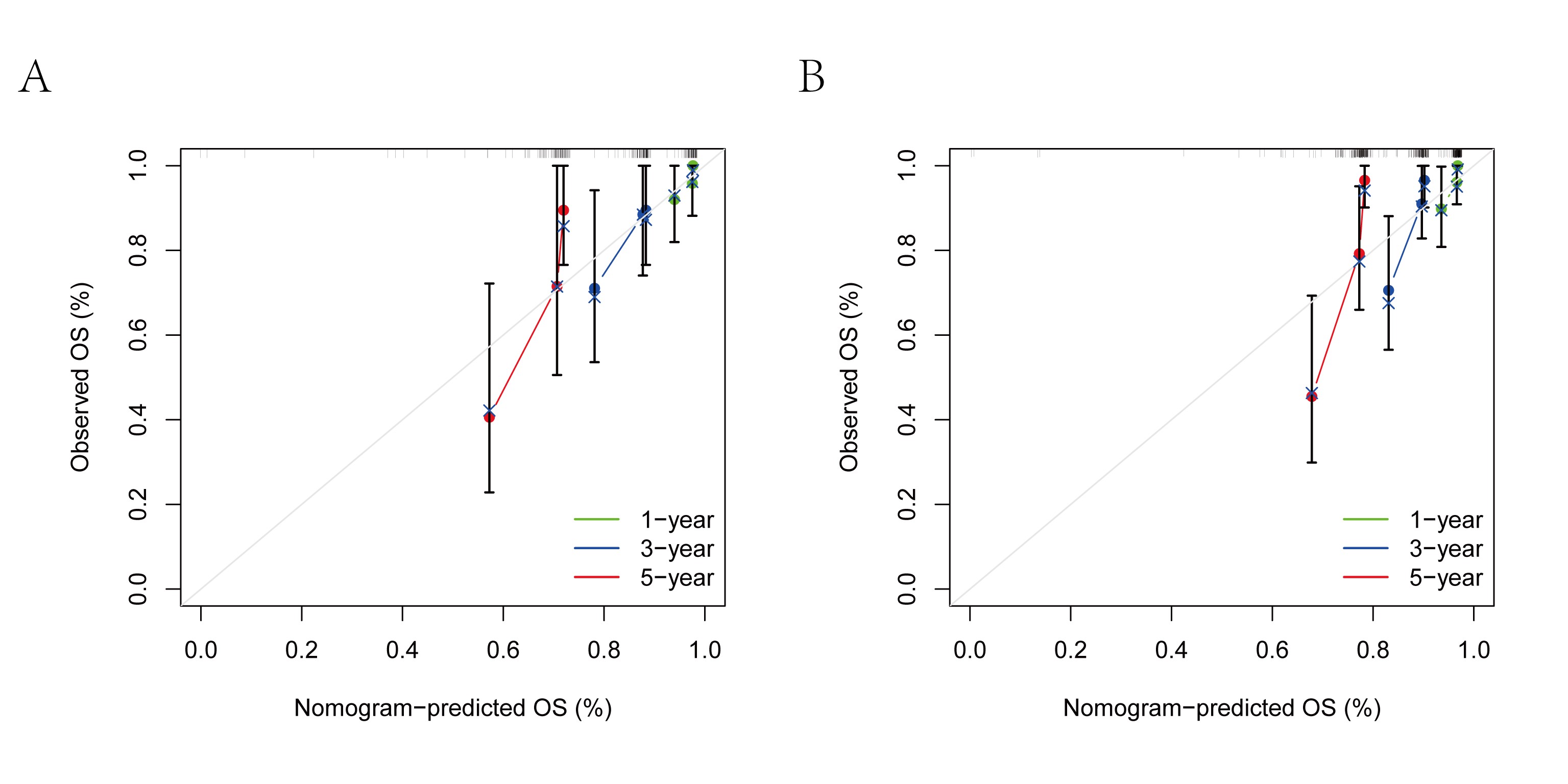

Supplement: Supplementary Figure 6 — The calibration curves of nomogram in the training cohort (A) and testing cohort (B). [file Image_6.jpeg]

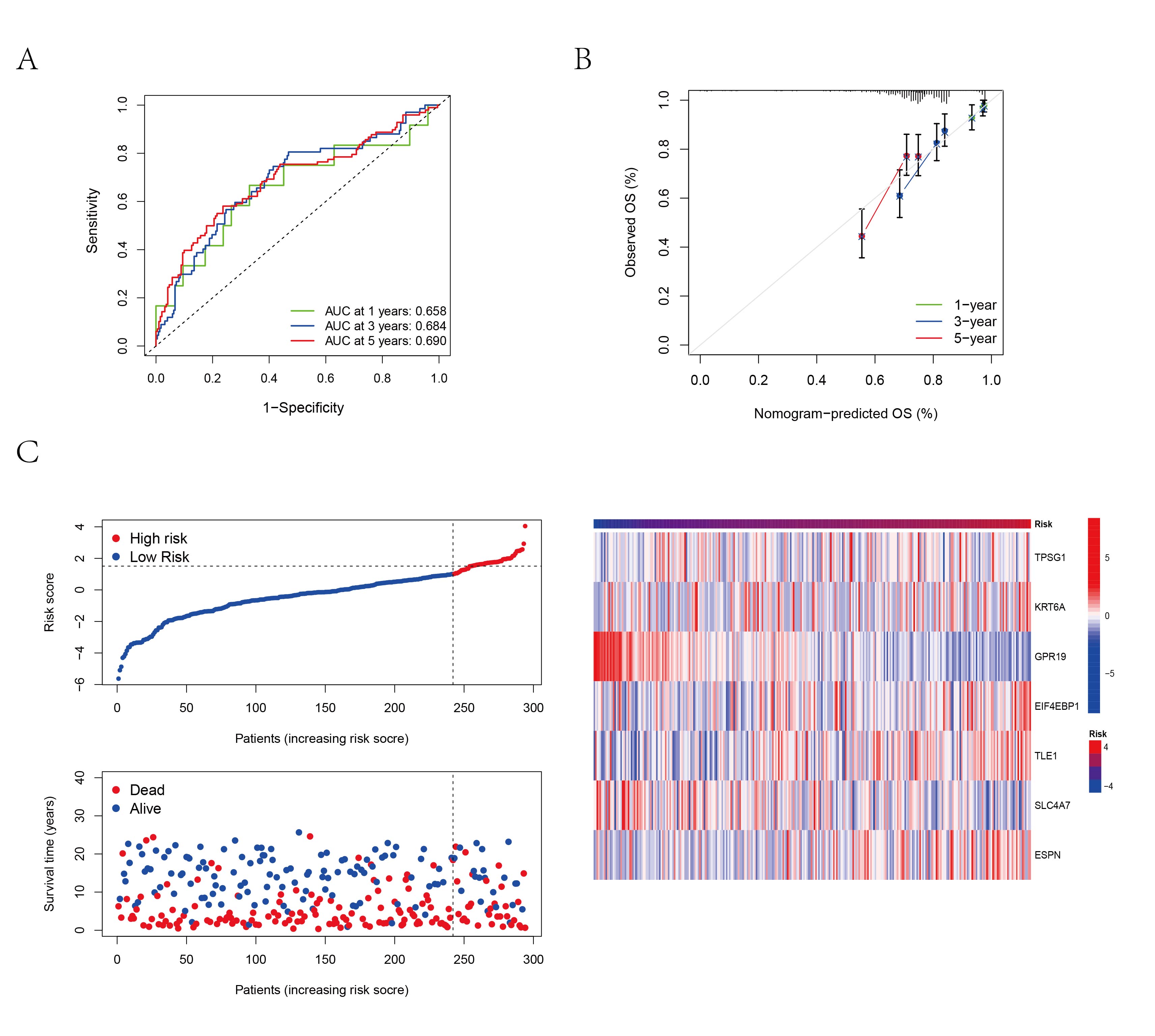

Supplement: Supplementary Figure 7 — The validation of nomogram in the Metabric database. (A). ROC curves to predict 1-, 3-, and 5-year OS according to the nomogram; (B). The calibration curves of nomogram. (C). Ranked dot, scatter plots, and heatmap showing the risk distribution, patient survival status and gene expression. OS, overall survival. [file Image_7.jpeg]
